# Characterization of the complete chloroplast genome of *Salix variegata* (Salicaceae)

**DOI:** 10.1080/23802359.2019.1698989

**Published:** 2019-12-12

**Authors:** Juan Chen

**Affiliations:** Key Laboratory of Eco-environments in Three Gorges Reservoir Region (Ministry of Education), Chongqing Key Laboratory of Plant Ecology and Resources Research in Three Gorges Reservoir Region, School of Life Sciences, Southwest University, Chongqing, PR China

**Keywords:** *Salix variegata*, Salicaceae, chloroplast genome, phylogenetic tree

## Abstract

The complete chloroplast genome sequence of *Salix variegata*, a native shrub willow species in the south of China, is reported. The plastome is 155,743 bp in length, with one large single copy region of 84,950 bp, one small single copy region of 16,219 bp, and two inverted repeat (IR) regions of 27,287 bp. It contains 130 genes, including 83 protein-coding genes, eight ribosomal RNA, and 38 transfer RNA. Phylogenetic tree shows that this species is a sister to *S. purpurea*. The published plastome within *Salix* provides significant insight for elucidating the phylogenetic relationship of taxa within Salicaceae.

Salicaceae (named willow family), a family of dioecious catkin-bearing woody plants, are well known for their worldwide diverse uses including willows (*Salix*) and poplars (*Populus*) (Fang et al. 1999; Ohashi, 2001). *Salix variegata* Franch., a native shrub willow species in the south of China (Fang et al. 1999). In our study, we characterized the complete chloroplast genome sequence of *S. variegata.* As one of important target for genetic transformation, the full chloroplast genome could supply more genetic information.

Fresh leaves of *S. variegata* were collected from Xiaoyudong town (Pengzhou, Sichuan, China; coordinates: 103°45′E, 31°11′N) and dried with silica gel. The voucher specimen was stored in Sichuan University Herbarium with the accstion number of Zl20190705001. Total genomic DNA was extracted with a modified CTAB method (Doyle and Doyle 1987). First, we obtained 10 million high quality pair-end reads for *S. variegata*, and after removing the adapters, the remained reads were used to assemble the complete chloroplast genome by NOVOPlasty (Dierckxsens et al. 2017). The complete chloroplasts genome sequence of *S. purpurea* was used as a reference. Plann v1.1 (Huang and CronK 2015) and Geneious v11.0.3 (Kearse et al. 2012) were used to annotate the chloroplasts genome and correct the annotation.

The total plastome length of *S. variegata* (MN698825) is 157,743 bp, exhibits a typical quadripartite structural organization, consisting of a large single copy (LSC) region of 84,950 bp, two inverted repeat (IR) regions of 27,287 bp, and a small single copy (SSC) region of 16,219 bp. The cp genome contains 130 complete genes, including 83 protein-coding genes (83 PCGs), eight ribosomal RNA genes (four rRNAs), and 38 tRNA genes (38 tRNAs). Most genes occur in a single copy while 17 genes occur in double, including all rRNAs (4.5S, 5S, 16S, and 23S rRNA), seven tRNAs (trnA-UGC, trnI-CAU, trnI-GAU, trnL-CAA, trnN-GUU, trnR-ACG, and trnV-GAC), and six PCGs (rps7, rps19, rpl2, rpl23, ndhB, and ycf2). The complete ycf1 gene was located at the SSC/IRa junction as a pseudogene. The overall AT content of cp DNA is 63.3%, the corresponding values of the LSC, SSC, and IR regions are 65.6%, 69.0%, and 58.0%.

In order to further clarify the phylogenetic position of *S. variegata*, plastome of 15 representative Salicaceae species were obtained from NCBI to construct the plastome phylogeny, with *Itoa orientalis* as an outgroup. All the sequences were aligned using MAFFT v.7.313 (Katoh and Standley 2013) and maximum-likelihood phylogenetic analyses were conducted using RAxML v.8.2.11 (Stamatakis 2014). The phylogenetic tree shows that *Salix* clade was identified two subclades. *S. paraplesia*, *S. babylonica S. chaenomeloides*, and *S. interior* together one clustered. Remian *Salix* species together another clustered while *S. variegata*, *S. purpurea* clustered together with *S. suchowensis* in this subclade (Figure 1).

**Figure 1.**
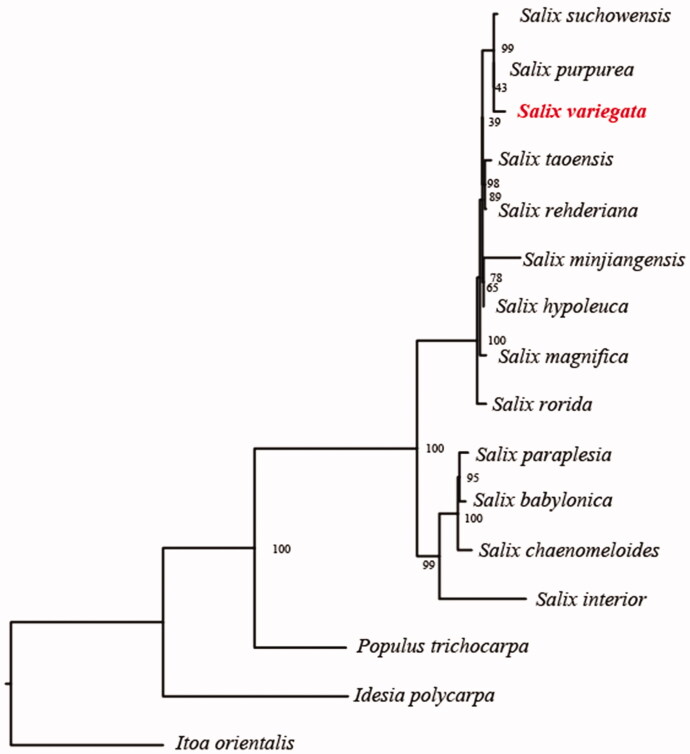
Phylogenetic relationships of Salicaceae species using whole chloroplast genome. GenBank accession numbers: *Salix suchowensis* (NC_026462), *Salix purpurea* (NC_026722), *Salix taoensis* (MG262369), *Salix rehderiana* (MG262367), *Salix minjiangensis* (MG262365), *Salix hypoleuca* (MG262363), *Salix magnifica* (MG262364), *Salix rorida* (MG262368), *Salix paraplesia* (MG262366), *Salix babylonica* (MG262361), *Salix chaenomeloides* (MG262362), *Salix interior* (NC_024681), *Populus trichocarpa* (NC_009143), *Idesia polycarpa* (NC_032060), and *Itoa orientalis* (MG262342).
